# A successfully treated case of herpes simplex encephalitis complicated by subarachnoid bleeding: a case report

**DOI:** 10.1186/1752-1947-4-310

**Published:** 2010-09-22

**Authors:** Yasuyo Tonomura, Hiroshi Kataoka, Noritaka Yata, Makoto Kawahara, Kazuo Okuchi, Satoshi Ueno

**Affiliations:** 1 Department of Neurology, Nara Medical University, 840 Shijo-cho, Kashihara, Nara 634-8522, Japan; 2 Department of Emergency and Critical Care Medicine, Nara Medical University, Kashihara, Nara, Japan

## Abstract

**Introduction:**

Histopathologically, herpes simplex virus type 1 causes hemorrhagic necrosis. Overt hemorrhage is infrequent in herpes simplex virus encephalitis but can lead to poor outcomes. This report describes a successfully treated case of herpes simplex virus encephalitis associated with subarachnoid bleeding in which real-time polymerase chain reaction was useful for diagnosis.

**Case presentation:**

A 30-year-old previously healthy Japanese woman who had fever and headache for five days presented with disorganised speech, unusual behavior and delusional thinking. Real-time polymerase chain reaction amplification of herpes simplex virus type 1 in cerebrospinal fluid was positive (38,000 copies/mL) and antivirus treatment was started. During the course of her illness, the level of her consciousness decreased in association with desaturation and tachycardia. Thrombosis of the right pulmonary artery trunk with pulmonary embolism was evident on enhanced chest computed tomography. In addition, cranial computed tomography revealed subarachnoid and intraventricular bleeding. Intravenous heparin (12,000 U/day) was started and the dose was adjusted according to the activated partial thromboplastin time for about a month (maximum dose of heparin, 20,400 U/day). After the treatments, her Glasgow coma score increased and the thrombosis of the pulmonary artery trunk had disappeared.

**Conclusions:**

The present case raises the question of whether anticoagulant treatment is safe in patients with herpes simplex virus encephalitis complicated by subarachnoid bleeding.

## Introduction

Herpes simplex virus type 1 (HSV) can cause fatal sporadic encephalitis in humans. Despite treatment, the mortality rate remains high, ranging from 20% to 30% [1]. Histopathologically, HSV causes hemorrhagic necrosis [2]. Overt hemorrhage is infrequently seen in HSV encephalitis (HSVE) but can lead to poor outcomes. We describe a successfully treated case of HSVE associated with subarachnoid bleeding in which real-time polymerase chain reaction (PCR) was useful.

## Case presentation

A 30-year-old previously healthy Japanese woman, who had fever and headache for five days, presented with disorganized speech, unusual behavior and delusional thinking. After two days, the level of consciousness decreased and the patient was admitted to our hospital.

**Figure 1:**
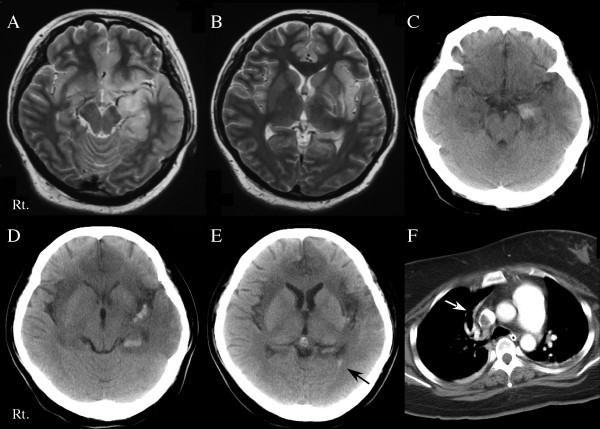
**Cranial T2-weighted magnetic resonance imaging (panel A and B) showed left-predominant bilateral regions of increased signal intensity in the hippocampus and amygdaloid body, the insular, medial temporal and medial frontal lobes**. Cranial computed tomography (CT; panel C) demonstrated high intensity lesions in the left amygdaloid body. Subarachnoid and intraventricular bleeding, in addition to low intensity lesions in the bilateral frontal and temporal lobes, was evident (panels D and E). A chest-enhanced CT demonstrated massive thrombosis of the right pulmonary artery trunk (panel F).

She was comatose and had a fever (39.1°C). The Glasgow coma score (GCS) was 7: eye opening, verbal response and motor response were 1, 2 and 4, respectively. Meningismus was present. Her eyeballs deviated to the left; the pupils were equal and normally reactive to light. The deep tendon reflexes were normal, with no pathological reflex. As she had frequently experienced generalized seizures with hypoventilation, the patient received mechanical ventilation. Intravenous sedation (midazolam) was started. The white cell count was 18200/μL and the C-reactive protein concentration was elevated (13.5 mg/dL). Other blood cell counts and the results of routine biochemical analysis were normal. Cranial T2-weighted magnetic resonance imaging showed bilateral regions of increased signal intensity in the hippocampus and amygdaloid body, the insular, medial temporal and medial frontal lobes (Figure 1A and 1B). A lumbar puncture on day one showed 321 white cells/mm^3 ^(93% lymphocytes, 7% polyneutrophils), 1 red cell/mm^3^, a protein concentration of 66 mg/dL and a glucose concentration of 74 mg/dL. Real-time PCR amplification of HSV-1 in cerebrospinal fluid (CSF) was positive (38,000 copies/mL). HSV-1 immunoglobulin M (IgM) and immunoglobulin G (IgG) antibodies were not detected in the CSF. In the serum, HSV-1 IgM antibodies were absent and the HSV-1 IgG antibody titer was 26.3. HSVE was diagnosed.

The patient received intravenous acyclovir (10 mg/kg/day, 10 days), dexamethasone (16 mg/day, five days) with tapering and immunoglobulin (5 g/day, three days). Anticonvulsant treatment with phenytoin (250 mg/day), valproate (900 mg/day) and phenobarbital (100 mg/day) was also begun. As she developed a fever (body temperature of over 40°C), her body temperature was lowered using a forced-air-cooling blanket. Her core temperature was maintained at between 36°C and 37°C for nine days.

Cranial computed tomography (CT) performed on day five showed hemorrhagic foci in the left amygdaloid body and low-intensity bilateral lesions in the frontal and temporal lobes. We performed repeated lumbar punctures in order to evaluate the disease severity and the responses to these treatments because a reduced consciousness level and cranial neuroimaging abnormalities persisted. CSF analysis performed on day seven showed 188 lymphocytes/mm^3^, 38 red cells/mm^3^, a glucose concentration of 72 mg/dL and increased titers of HSV-1 IgM and IgG antibodies (3.08 and 6.17, respectively).

On day 11 after admission, the results of real-time PCR for HSV-1 in CSF were negative, but CSF lymphocytes and red cells had increased to 189/mm^3 ^and 125/mm^3^, respectively, and intracranial hemorrhage was clearly evident (Figure 1C). The glucose concentration in CSF was 79 mg/dL. Antiviral treatment was switched from acyclovir to intravenous vidarabine (900 mg/day, 14 days). At this time, HSV-1 IgM and IgG antibodies were 7.89 and 11.2, respectively, in the CSF and 0.56 and 76 in the serum.

On day 21, sedative medication and mechanical ventilatory support were withdrawn and the GCS increased to 9 (eye opening, verbal response and motor response were 3, 2 and 4, respectively).

On day 26, the level of consciousness decreased in association with desaturation and tachycardia. Thrombosis of the right pulmonary artery trunk with pulmonary embolism was evident on enhanced CT of the chest (Figure 1F). A high serum D-dimer persisted (maximum titer: 48.3 μg/mL). In addition, cranial CT revealed subarachnoid and intraventricular bleeding (Figure 1D and 1E).

During her hospitalization, she did not experience any intermittent or persistent hypertension. Intravenous heparin (12,000 U/day) was started and the dose was adjusted according to the activated partial thromboplastin time for about a month (maximal dose of heparin, 20,400 U/day). CSF analysis on day 39 showed 6 lymphocytes/mm^3^, 52 red cells/mm^3 ^and a glucose concentration of 78 mg/dL; the titers of HSV-1 IgM and IgG antibodies were 1.34 and greater than 12.8, respectively. Cranial CT on day 54 showed that the subarachnoid and intracranial bleeding had disappeared. Enhanced CT angiography demonstrated an avascular area in the left temporal lobe but no other arterial or venous abnormalities, such as aneurysm formation or irregular vascular distribution, were evident (data not shown).

Three months after admission, she responded to simple orders. Her GCS increased to 14 (eye opening, verbal response and motor response were 4, 5 and 5, respectively) and thrombosis of the pulmonary artery trunk had disappeared. As her consciousness level had reduced, informed consent for the above medical treatments and procedures was obtained from her family.

## Discussion

PCR has become the standard diagnostic test for HSVE. However, intrathecal antibody measurements are still of value, with an estimated specificity of 80% or 95% [3]. Real-time PCR is a recent modification of conventional PCR for HSV. The relation between the results of PCR and intrathecal antibody levels remains poorly understood. This issue has been addressed by one study but real-time PCR and measurement of antibody titers were performed in many patients at different times [4]. Intrathecal viral genomes on PCR and increased intrathecal HSV antibodies have been detected within five days [5] and after seven days [6] from the onset of neurologic symptoms, respectively. Our study found that the results of real-time HSV PCR were positive three days after the onset of central nervous symptoms, without intrathecally synthesized specific HSV antibodies.

Intracerebral hematoma is rarely associated with HSVE [7] and only 14 cases have so far been reported. To the best of our knowledge, this is the first report to document a case of HSVE associated with subarachnoid bleeding. Obvious abnormalities of major cerebral vascular arteries, such as aneurysm formation and an irregular distribution of the anterior, middle and posterior cerebral arteries, were not evident which suggests that the subarachnoid bleeding was directly attributed to HSVE. HSV causes a necrotizing vasculopathy ascribed to cortical and subcortical intense hemorrhagic necrosis and perivascular cuffing in the medial temporal and orbitofrontal regions [2] and CSF analysis often demonstrates the presence of red cells. In gyri located near the CSF, diffuse necrotizing angiitis of venules and capillaries induced by intense inflammatory necrotizing vasculopathy [8] can cause vessel wall necrosis and subsequent bleeding, leading to hematogenous spread into the CSF space. Subarachnoid bleeding in our patient may have been caused by red-cell diapedesis from the hemorrhagic necrotizing amygdaloid body into the adjacent CSF spaces, resulting in 'subarachnoid bleeding with intraventricular extension'. Coagulopathy or hepatocellular damage with a consequent insufficient production of clotting factors can complicate severe HSV infections [9] and may potentially cause bleeding.

## Conclusions

Focal intense HSVE can increase the risk of subarachnoid bleeding and our experience raises the question of whether anticoagulant treatment is safe for patients with HSVE complicated by subarachnoid bleeding.

## Abbreviations

CSF: cerebrospinal fluid; CT: computed tomography; GCS: Glasgow coma score; HSV: herpes simplex virus type 1; HSVE: HSV encephalitis; IgG: immunoglobulin G; IgM: immunoglobulin M; PCR: polymerase chain reaction.

## Consent

Written informed consent was obtained from the patient for the publication of this case report and any accompanying images. A copy of the written consent is available for review by the Editor-in-Chief of this journal.

## Competing interests

The authors declare that they have no competing interests.

## Authors' contributions

YT, HK, MK, NY, KO and SU reviewed the existing literature and drafted the manuscript which was edited by HK. HK reviewed and selected radiology images. All authors read and approved the final manuscript.
